# An Electrochemical *o*-Phthalaldehyde Sensor Using a Modified Disposable Screen-Printed Electrode with Polyacrylate Hydrogel for Concentration Verification of Clinical Disinfectant

**DOI:** 10.3390/bios13040485

**Published:** 2023-04-17

**Authors:** Richie L. C. Chen, Bo-Chuan Hsieh, Jia-Sin Lin, Tzong-Jih Cheng

**Affiliations:** 1 Department of Biomechatronics Engineering, College of Bio-Resources and Agriculture, National Taiwan University, Taipei 10617, Taiwan; rlcchen@ntu.edu.tw (R.L.C.C.); hsiehpc@ntu.edu.tw (B.-C.H.); r03631020@ntu.edu.tw (J.-S.L.); 2 Department of Biomedical Engineering, National Taiwan University Hospital, College of Medicine, National Taiwan University, Taipei 10617, Taiwan

**Keywords:** disinfectant, *o*-phthalaldehyde (OPA), hydrogel, screen-printed carbon electrode (SPCE), electrochemical sensor

## Abstract

The study proposes an *o*-phthalaldehyde (OPA) sensor for rapid and reliable detection of OPA in healthcare disinfection practices, based on a hydrogel-modified screen-printed carbon electrode strip. The hydrogel film, which contains glycine and *N*-acetylcysteine, reacts with OPA to produce a reductive isoindole derivative. The derivative is then oxidized for OPA determination using cyclic voltammetry. The proposed sensor achieves an optimal detection time of 20–30 s and requires only a small analyte volume of 5 µL. It exhibits good precision (10%) and sensitivity (3.3 μA/cm^2^ mM) in a phosphate-buffered solution (pH 7.6), with excellent linearity (R^2^ > 0.97) and precision (<3%) in the detection range (0.2–0.6%) required for clinical OPA solutions. Moreover, the sensor demonstrates good concentration verification of Cidex-OPA disinfection in healthcare institutes, with high sensitivity (18.28 μA/cm^2^ mM) and precision around the minimum effective concentration (0.3%). Overall, the proposed sensor offers a promising and practical solution for accurate and reliable OPA detection in clinical disinfection practices.

## 1. Introduction

Sterilization and disinfection are crucial processes in healthcare that involve destroying or deactivating microorganisms, including bacterial spores, using physical or chemical means. Semi-critical devices such as endoscopes require high-level disinfection to remove all non-spore microorganisms [1]. Proper disinfectant use is essential in this process, as evidenced by previous studies [2,3]. The *o*-phthalaldehyde (OPA) is a disinfectant that has been used to disinfect endoscopes for over 14 days without the need for additional stabilization procedures [4,5,6,7]. This disinfectant has several advantages over others commonly used in healthcare settings, such as good material compatibility, lower toxicity, and effectiveness in disinfecting endoscopes and biosensors [8,9,10,11,12,13]. In contrast, disinfectants such as glutaraldehyde (GA), ethanol, hydrogen peroxide, and peracetic acid have various drawbacks, including irritation, flammability, material corrosion, and damage to sensitive materials. Notably, Cidex-OPA (0.55%) achieved effective disinfection without any detectable loss in the performance of electrochemical aptamer-based sensors [14].

To ensure the effectiveness of OPA disinfectant, it is crucial to verify its concentration before each disinfection process. In clinical settings, the minimum effective concentration (MEC) of OPA (0.3%) [15] is typically verified using commercially available indicator strips [16]. However, indicator strips are only semi-quantitative, and their accuracy is affected by time-limited test steps and store conditions such as temperature and humidity [17]. High-pressure liquid chromatography (HPLC) can precisely determine OPA concentrations, but its bulkiness, operational costs, and lack of portability limit its clinical applicability [15]. As such, there is a need for a more precise and quantitative OPA sensor that is portable and suitable for use in clinical disinfection processes.

Various methods for protein/peptide assay [18], amino-acid/amine determination [19], and thiols detection [20] have been utilized, including fluorescence assay [21], electrochemistry [22], and colorimetry (spectrophotometry) [23], to assess these analytes with OPA. High sensitivity fluorescence assays have been used by researchers, but they require expensive instrumentation. On the other hand, colorimetry is simple but crude. Electrochemical sensors have been preferred for their real-time measurements, high selectivity and specificity, and cost-effectiveness, making them ideal for a broad range of applications, including biomedical diagnostics, environmental monitoring, and food safety analysis. Therefore, an electrochemical method was employed in this work to realize an OPA sensor, using an amine and a thiol as the reactants. The most suitable method was chosen by considering trade-offs in assay performance, instrumental cost, and convenience.

This study aimed to develop a quantitatively electrochemical sensor and a meter capable of detecting disinfectant OPA’s MEC with high precision. The detection ranges specified were between 0.2 and 0.6%. These parameters were established based on clinical infection control, quality management, and cost requirements. An SPCE modified with PA-hydrogel containing glycine and N-acetylcysteine (NAC) was used to construct the electrochemical OPA sensor, and the oxidation current of the isoindole derivative of OPA, glycine, and NAC determined the OPA dose level. The study focused on validating the feasibility of the sensor in meeting the precision and process compatibility required for clinical disinfection needs, with a detailed investigation of the optimal conditions for developing the OPA sensor.

## 2. Materials and Methods

### 2.1. Experimental Instruments

Electrochemical measurements were conducted using a CHI Instruments 410C Electrochemical Analyzer from Tennison Hill Drive, Austin, TX, USA. The disposable screen-printed carbon electrodes (SPCEs) with a 3 mm diameter (TE100, Zensor Research & Development, Taichung, Taiwan) were used for the measurements. Additionally, the electrochemical sensing platform for measuring Cidex-OPA concentrations was based on the LMP91000EVM from Texas Instruments, Dallas, TX, USA, and was interfaced by an Arduino UNO from Italy. These measurements and sensing techniques were utilized to determine the concentrations of Cidex-OPA disinfectant for research purposes.

### 2.2. Reagents

The following chemicals were obtained from various suppliers: *o*-phthalaldehyde (OPA), disodium hydrogen phosphate, sodium dihydrogen phosphate, and sodium carbonate from Nacalai tesque (Kyoto, Japan); glycine and *N*-acetylcysteine (NAC) from Sigma (St. Louis, MO, USA); citric acid, sodium citrate, and sodium polyacrylate from Wako (Osaka, Japan); Triton X100 and ethanol (95%) from Sigma (St. Louis, MO, USA); alcohol from Shimakyu’s Pure Chemicals (Osaka, Japan); and hydrochloric acid from Union Chemical Works Ltd. (Taichung, Taiwan). All analytical-grade reagents were used without further purification. Ultra-pure water with a resistivity greater than 18.2 MΩ cm was prepared using a Direct-Q gradient system (Millipore, Milford, MA, USA).

Buffers were prepared by dissolving the following chemicals in deionized water and storing them at room temperature as the stock: borate buffer (0.1 M, pH 8–10 with NaOH), phosphate buffer (0.1 M, pH 6–8 with NaOH), and citrate buffer (0.1 M, pH 4–6 with NaOH). The 20% OPA reagent was prepared by mixing 2 g of OPA in 10 mL of 99% ethanol. The 1% OPA reagent was freshly prepared by dissolving 0.1 g OPA in 10 mL of ultra-pure water and then diluting it to 0.6, 0.5, 0.4, 0.3, and 0.2%.

### 2.3. Anodic Pretreatments of SPCEs

The study compares the repeatability and reproducibility of three anodic treatment methods for the SPCE electrode. The methods are as follows: (1) immerse the SPCE in a 0.05 M pH 7.4 phosphate buffer solution and apply a voltage of +1.2 V at a sweep rate of 100 mV/s for 2 min [24]; (2) immerse the SPCE in a saturated sodium carbonate solution and apply a voltage of +1.2 V at a sweep rate of 60 mV/s for 5 min [25]; and (3) immerse the SPCE in 3 M NaOH solution for 1 h, place the electrode in 0.5 M NaOH solution, and apply a voltage of +1.2 V at a sweep rate of 10 mV/s for 20 s [26].

For the repeatability test, the amperometry was used to detect 4 mM ferrocyanide in a 1 M potassium chloride solution, and each of the three anodized electrodes was tested three times. For the reproducibility test, three electrodes were taken from each of the three anodized methods for the quintuplet test, and the variation coefficients of the three methods were compared.

### 2.4. Preparation of OPA Sensors

Before hydrogel modification, the electrode surfaces of the SPCEs were pre-treated to the optimal condition obtained from the above-mentioned part to be electrochemically cleaned. They were then stored dry at room temperature before use. The glycine stock solution was prepared with glycine (200 mM) mixed with TX100 (7.5%) by dissolving in a 0.1 M pH 7.6 phosphate buffer. The NAC/PA stock solutions were prepared by dissolving sodium polyacrylate (50%) and NAC (120 mM) in a 0.1 M pH 7.6 phosphate buffer. The modification area was defined by covering the designated surface area of the SPCEs with insulating tape. A total of 5 μL of glycine stock solution was firstly applied onto the modification area of SPCE and then left to dry at room temperature. Next, the hydrogel-modified electrochemical strips were covered with 5 μL of NAC/PA stock solution and then stored at room temperature until dry.

### 2.5. Response Time of Hydrogel-Modified SPCEs

The experiment was conducted by covering the hydrogel-modified electrode surface completely with 5 µL of a 0.5% OPA solution. The response time required for the chemical oxidation of OPA to diffuse into the hydrogel and then to the electrode surface was determined by using the peak current of CV. The response of the sensor was recorded every 20 s until the current peak was no longer significantly changing.

### 2.6. Function and Performance of Hydrogel-Modified SPCEs

To confirm that the disinfectant could be quantified by the hydrogel-modified electrode, amperometry was performed with the hydrogel-modified SPCEs to determine the 0.2, 0.3, 0.4, 0.5, and 0.6% reference OPA. The SPCE was covered with 5 μL of OPA reagent for 20 s and then subjected to +0.8 V to obtain the current response at 5 s. Triplet tests were performed for all conditions.

For comparison with the reference OPA solutions, the commercially available disinfectant Cidex-OPA reagent was diluted to the required concentrations. The hydrogel-modified SPCE strips were used to verify the performance of the original 0.55% concentration of Cidex-OPA and diluted 0.25, 0.30, 0.35, and 0.45% solutions, with the disinfection MEC (0.3% Cidex-OPA) being the necessary condition for experimental designs.

### 2.7. Determination of Cidex-OPA by Commercial OPA Test (Indicator) Strips

The test strip was submerged into the disinfectant solution being tested and removed after one second. The strip was then stood upright on a paper towel to remove excess solution from the indicating pad. The results of the color reaction on the indicating pad were recorded by taking a photo 90 s after the test strip was removed from the solution.

## 3. Results

### 3.1. Cyclic Voltammetric Characterization

**Figure 1:**
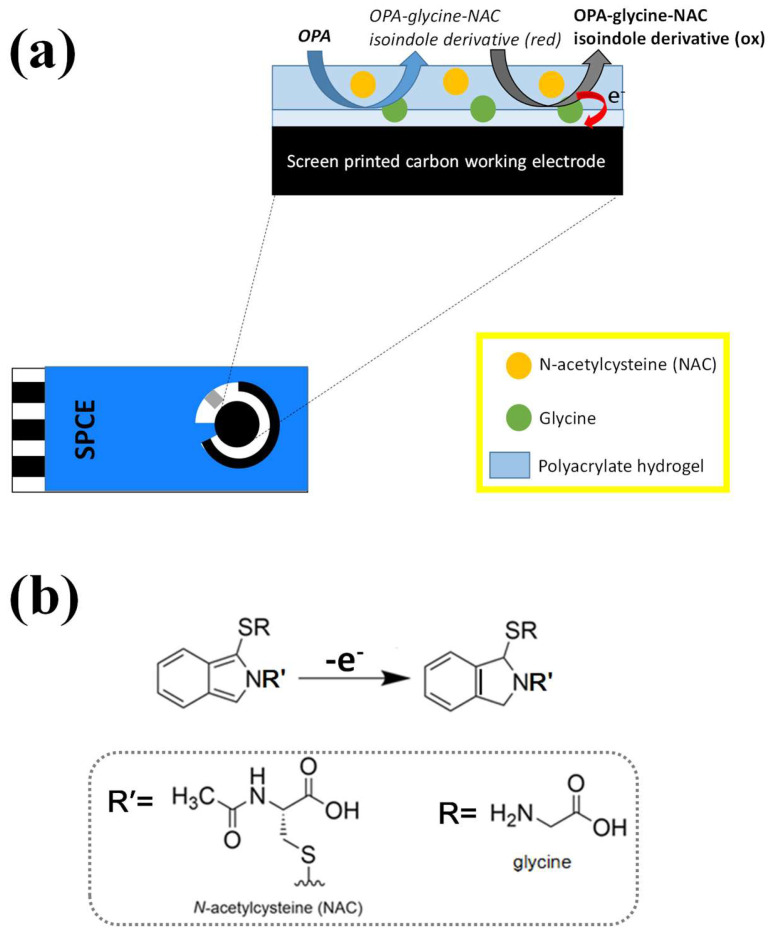
The principle (**a**) and a possible oxidation reaction (**b**) of an electrochemical OPA sensor with a hydrogel-modified SPCE strip are shown in a schematic diagram. When the surface of the modified electrode is dropped with the OPA analyte, it first reacts with NAC on the outer layer to form an OPA-NAC complex. Then, the complex is diffused to the inner layer to react with glycine to create a reduced OPA-glycine-NAC isoindole derivative. The derivative is electrochemically oxidized by applying an appropriate voltage to the electrode, and the resulting oxidation current is used to quantitatively determine the concentration of OPA.

A schematic overview of the electrochemically modified hydrogel sensor is shown in Figure 1. The sensing hydrogel was constructed on SPCE using a two-step preparation procedure in this study. Stable electrochemical responses were observed with pre-adsorption of glycine for developing NAC-containing hydrogel in our preliminary pilot work. The polyacrylate (PA) hydrogel film modified on SPCEs was formatted with the reductive OPA-glycine-NAC isoindole derivative. The isoindole derivative was then electrochemically oxidized on the electrode surface and applied with sufficient potential. The electrochemical characteristics of glycine, NAC, OPA, and OPA-glycine-NAC isoindole derivatives were investigated individually by bare SPCEs in phosphate buffer (0.1 M pH 7.0).

**Figure 2:**
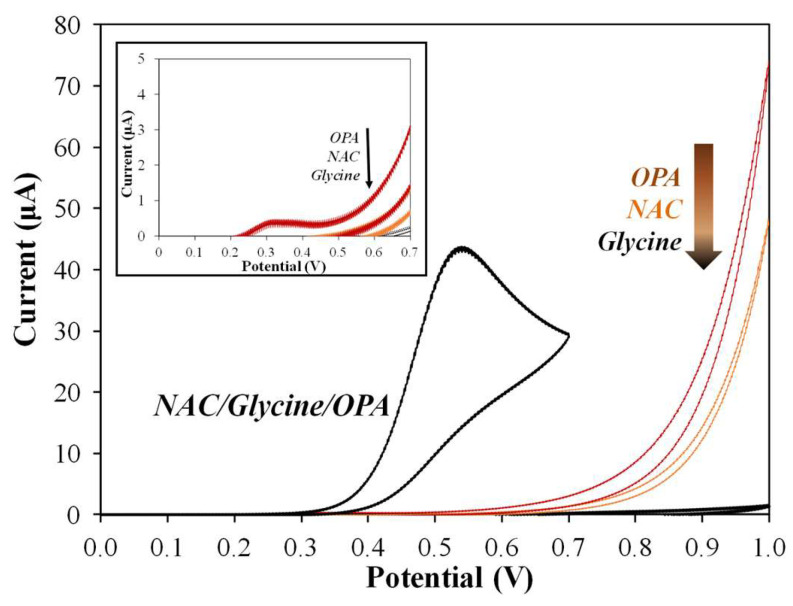
Cyclic voltammograms of glycine (5 mM), NAC (5 mM), OPA (0.1%), and their mixtures were measured using bare SPCEs in 0.1 M PBS (pH 7.0) via cyclic voltammetry with a scan rate of 100 mV/s. The applied potential was measured relative to Ag/AgCl. A zoom-in diagram was included to show the output range of 0–5 μA.

Figure 2 shows a well-defined oxidative current peak for the OPA-glycine-NAC isoindole derivative at a potential of +0.55 V versus Ag/AgCl, while almost no electrochemical response was observed for individual OPA, NAC, or glycine in the potential range from 0 V to +0.7 V. These findings are consistent with previous research reports indicating that OPA chemically reacts with glycine and NAC to form OPA isomers [27]. The CV response profile and peak current were also consistent with a report investigating the redox chemistry of isoindoles [28]. The electrochemical sensing principle of OPA used in this work was demonstrated by the specific response to the OPA analyte with the reactant containing glycine and NAC.

### 3.2. Acidic Effect on Odixation of the OPA-Glycine-NAC Isoindole

**Figure 3:**
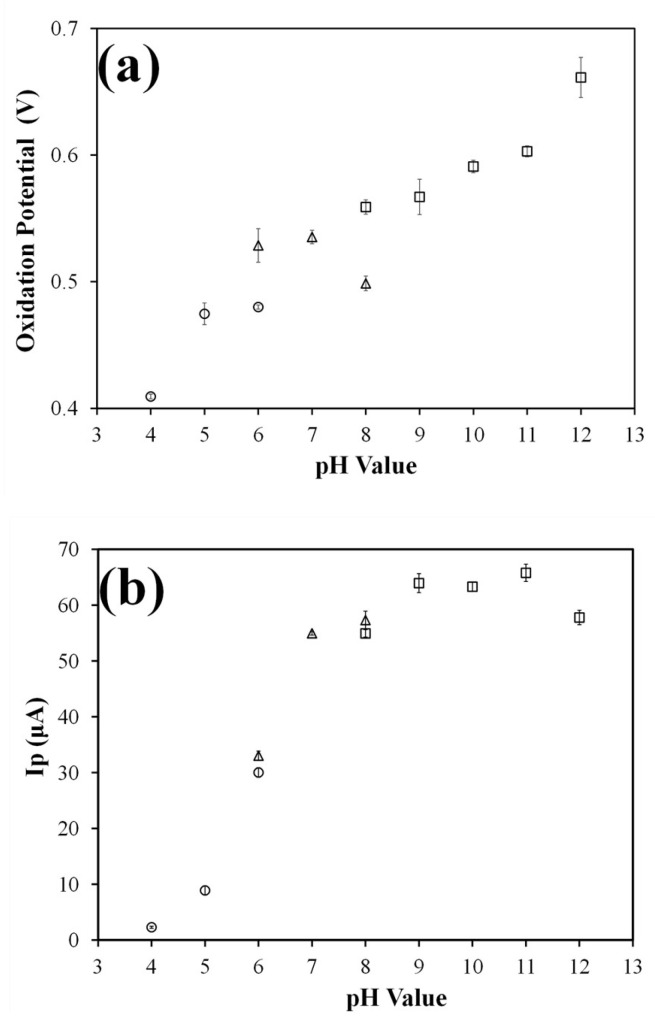
The effect of acidity on the oxidation potential and current of OPA-glycine-NAC isoindole. Cyclic voltammograms were obtained by bare SPCEs with a scan range of 0 ± 0.7 V and a scan rate of 100 mV/s. The solutions used for the experiment contained 5 mM glycine, 5 mM NAC, and 0.1% OPA in 0.1 M citrate buffer (pH 4–6) (○), 0.1 M PBS buffer (pH 6–8) (△), and 0.1 M borate buffer (pH 8–12) (□). The oxidation potentials and currents (I_p_) are the anodic peak potential and the anodic peak current, respectively, obtained from the same cyclic voltammogram. A typical CV is the same as the NAC/Glycine/OPA shown in Figure 2. Moreover, every experiment condition was performed in triplicate (n = 3).

Different pH conditions were tested to optimize the acidity of the detection medium for the electrochemical oxidation of OPA’s isoindole derivative. The oxidation potential of the isoindole product increased with increasing pH values, as shown in Figure 3a. Anions in solution had a slight effect on the oxidation potential, with the citric acid buffer having the best electron transfer efficiency, followed by the phosphate buffer and the borate buffer. The effect of anion species on the oxidation current was not apparent (Figure 3b). The peak value of the oxidation current increased with increasing pH value, reaching a maximum response under neutral and alkaline conditions (pH > 7). The formation of the isoindole derivative was relatively complete, with a pH value > 7, indicating a higher response to the analyte determination. Based on the balance between oxidation voltage and oxidation current optimization, we selected a 0.1 M phosphate buffer solution with a pH of 7.6 as the experimental electrolyte condition.

### 3.3. Optimization of the Glycine and NAC Concentration to OPA

**Figure 4:**
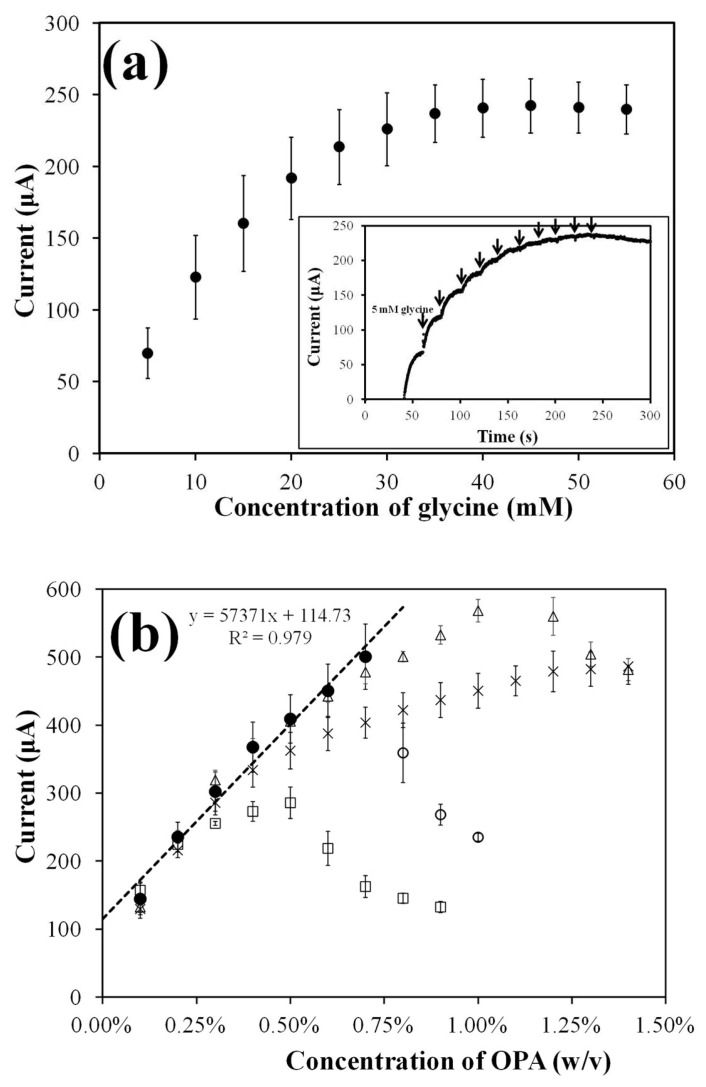
Optimization of the glycine and NAC concentrations to 0.6% OPA. (**a**) A calibration curve and an amperometric response (inset curve) were obtained for successive glycine additions in 0.1 M PBS (pH 7.6) containing 0.6% OPA and 80 mM NAC with bare SPCE at +0.7 V. (n = 3) (**b**) Calibration curves obtained for optimization of the NAC. The curve represents amperometric response for successive 0.1% OPA additions in 0.1 M PBS (pH 7.6) containing 50 mM glycine and (□) 20 mM, (○, ●) 30 mM, (△) 50 mM, and (×) 80 mM NAC with bare SPCE at +0.7 V. (n = 3). The experiment was carried out with sufficient stirring.

To ensure determination of the highest concentration of OPA in clinical applications, the maximum concentration of the analyte was preset at 0.60% OPA. As shown in Figure 4a, the oxidation current increased with the increase in glycine concentration under 80 mM NAC. Finally, the maximum value was reached, and the oxidation current tended to be saturated when the glycine concentration was higher than 40 mM.

Figure 4b shows the OPA calibration curve formed by different concentrations of NAC (20–80 mM) under 50 mM glycine. A significant decrease in oxidation current was observed in the presence of excessive OPA. For the reagent formulations containing 50 mM glycine and 20, 30, or 50 mM NAC, the concentrations of OPA that cause a decrease in oxidation current were found to be greater than 0.6%, 0.7%, and 1.0%, respectively. This observation is consistent with previous studies [29,30], which attribute the rapid degradation of isoindole derivatives to excess OPA. It should be noted that an excess NAC/glycine ratio of 80 mM/50 mM significantly reduces the oxidation current at high OPA concentrations (>0.6%). This inhibitory effect may be due to the reduction in the electrochemically active OPA-glycine-NAC isoindole derivative caused by the production of more OPA-NAC products by NAC and excess OPA. Previous research results [31,32] can help to understand the rapid degradation of isoindole derivatives caused by OPA. In summary, the reagent formula with equal concentrations of glycine and NAC shows a similar response at non-excessive OPA concentrations (<0.6%). However, the formula with a higher NAC/glycine concentration ratio has a significant response inhibition effect on determining high-concentration OPA. The former is more valuable for the application context of this research.

In the concentration range of 0.10~0.70% OPA, both 30 mM NAC and 50 mM NAC showed similar optimal sensitivity. The detection range specification was widened and set at 0.20–0.60%, which could meet the detection requirements of 0.6% OPA demand with 50 mM glycine and a concentration greater than 30 mM NAC. Due to the rapid Schiff base reaction between the amine and the aldehyde groups, glycine and OPA should not be placed in the same solution alone to avoid a direct response between glycine and OPA. The reaction reagent should be fully pre-mixed with glycine and NAC and then reacted with OPA-containing samples for testing.

### 3.4. Response Stability of the OPA-Glycine-NAC Isoindole

**Figure 5:**
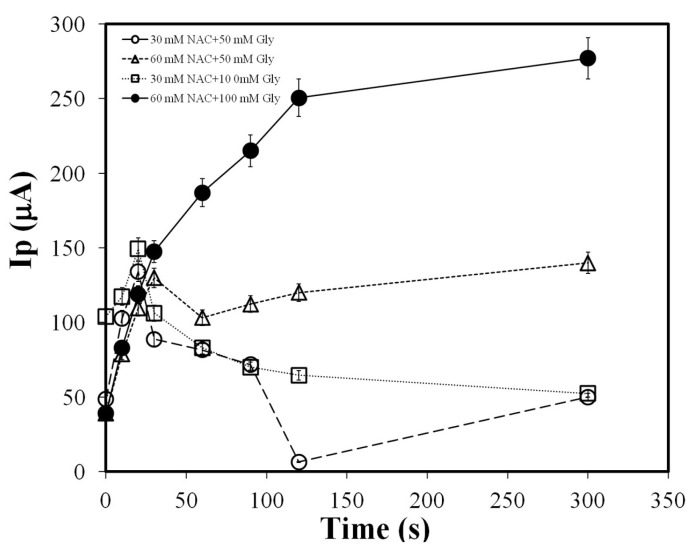
The response stability of the reagent compositions (glycine and NAC). Curves represent temporal dependence of the peak current of cyclic voltammograms for various compositions, including (○) 30 mM NAC/50 mM glycine, (△) 60 mM NAC/50 mM glycine, (□) 30 mM NAC/100 mM glycine, and (●) 60 mM NAC/100 mM glycine in the presence of 0.6% OPA with bare SPCE at +0.7 V. (n = 3).

Previous literature has pointed out that if the concentration of OPA solution is high, the stability of the isoindole derivative will be poor [31,33], which may not be conducive to precision measurement. Therefore, the stability of the reagents (glycine and NAC) reactions to the test object OPA was studied here. As shown in Figure 5, when the reagent composed of 50 mM glycine and 30 mM NAC reacts with 0.6% OPA, the current response drops to 3/5 of the maximum response after about 20–30 s, and the color of the reagent turns from colorless to black within a few seconds. As the glycine concentration is doubled (100 mM), there is no significant difference in the current response. Still, the color of the reagent changes from colorless to yellow within a few seconds. This color is related to the reaction of glycine and OPA. The product’s color is the same, so it can be inferred that excess glycine will react with OPA in the Schiff base, but it will not contribute to the electrochemical response stability of indole derivatives. As the NAC concentration is doubled (60 mM), its current response declines only slowly after about 30 s, and no apparent rapid decline is observed within 5 min. The reagent changes from colorless to pinkish purple, and its color will become more pronounced with time. Under the 100 mM glycine and 60 mM NAC conditions, 0.6% OPA was measured. Within 5 min, no decrease in the current response was observed, and the reagent remained transparent and colorless. However, its long response time (>120 s) hinders its development for time-critical applications.

By increasing the concentration of NAC, the stability of the oxidation current response of the derivative can be improved. Under the condition of a high concentration of NAC, although the increase in glycine concentration can improve the current response, it will significantly prolong the response time. For applications where the time required for the determination is not critical, the combination of 60 mM NAC and 50–100 mM glycine can provide a stable output response. In situations where rapid detection is required (<30 s), the reagent formulation combination of 30 mM NAC and 50 mM glycine is sufficient. Preliminary experimental results indicate that the reagent with 50 mM glycine and 30 mM NAC in 0.1 M PBS (pH 7.6) had a sufficient response to meet the requirements of the OPA sensor’s specifications, based on the upper detection limit of 0.6%. Furthermore, there should be a test procedure with precise time control to ensure good repeatability of the system.

### 3.5. Precision Improvement by Anodic Pre-Treatment of SPCEs

The use of inorganic adhesives or insulating polymers in the process of screen-printing electrodes results in an increase in impedance and variability of electron transfer on the surface of the substrate. To reduce variability in commercially available SPCEs, Table 1 presents the reproducibility and repeatability of SPCEs after different anodic pretreatments. The results suggest that the best method for reproducibility and repeatability is immersion of the electrode in a saturated sodium carbonate solution with an applied voltage of +1.2 V and a sweep rate of 60 mV/s for 5 min. It is inferred that microbubbles generated on the surface of the electrode during this process can effectively remove the adhesive on the surface of carbon particles, stabilize the surface of the electrode, and increase oxygen-containing functional groups.

**Figure 6:**
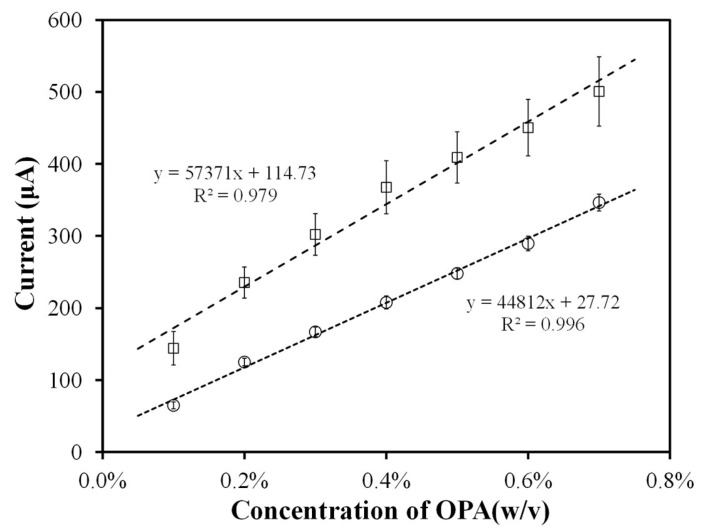
Calibration curve comparison between bare SPCE (□) and pretreated SPCE (○). Electrochemically pre-treated SPCEs were activated in saturated Na_2_CO_3_ at +1.2 V for 5 min. Batch determination of OPA by amperometry (at +0.7 V) in 0.1 M PBS (pH 7.6) containing 50 mM glycine/30 mM NAC, with the concentration ranging from 0.2% to 0.6% (*w*/*v*) OPA, n = 3.

To compare the reproducibility of the electrode pretreated with saturated sodium carbonate, Figure 6 shows OPA calibration curves obtained at the specified detection range (0.20–0.60% (*w*/*v*)). It is observed that the correlation coefficient of the electrode pretreated with saturated sodium carbonate increased from 0.979 to 0.996 and its variance decreased from 9.2% to 3%, demonstrating that the pretreatment procedure improves the reproducibility of quantifying OPA concentration. Moreover, electrochemical pretreatment of the electrode surface on the SPCE reduces variation in detection results, allowing the development of a precise threshold indicator with a narrow detection range. Therefore, the first procedure in the standard production procedure for making OPA sensors in the future will be the anodization of SPCEs with saturated sodium carbonate.

### 3.6. Response Time of the Hydrogel-Modified Electrochemical Strips

A hydrogel-modified electrode for electroanalysis is the basis of the proposed OPA sensor. The response amplitude and signal stability of the oxidative current of the sensor are affected by the amount of reagent consumed in the hydrogel (or the residual amount after consumption) and the time for the analyte to diffuse into the reagent-containing hydrogel film. The appropriate acquiring time for representatively sensing current signals was determined by investigating the response time of the OPA sensor using cyclic voltammetry.

**Figure 7:**
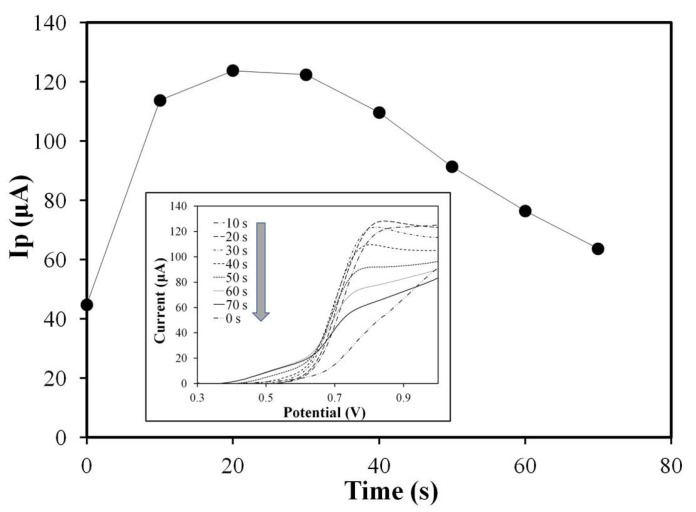
Temporal response characteristics of the OPA sensor. Time dependence of peak current (I_p_) responses of hydrogel-modified SPCE strips to 5 μL of 0.5% OPA. Insert shows cyclic voltammograms of the OPA sensors at various starting times (0–70 s) of the cyclic voltammetry scans. Scan rate: 100 mV/s.

It was found that diffusion-limited responses to the electrochemical OPA sensors were offered by cyclic voltammograms, as presented in the insert of Figure 7. The current peak varied with the time interval from dropping the analyte sample on the strip to starting the CV test. The relationship between the peak current (I_p_) of the OPA sensor and the time interval from dropping the analyte to starting the CV test is shown in Figure 7. The oxidation current peak value increased with the preconditioning time and reached the maximum value at t = 20 s in the initial phase (0–20 s), implying that sufficient time is required to form the isoindole derivative and/or its diffusion from the hydrogel to the electrode surface. In the second phase (20–30 s), the current response reaches its maximum value and maintains a consistent level for at least 10 s.

In the final phase (>30 s), the I_p_ response gradually decreased with the increase in the preconditioning time to adjust, which was consistent with the test results in the batch by the bare electrode (refer to Section 3.4). The same electrochemical response and temporal stability in batch tests (Section 3.4) and hydrogel modification tests (here) were exhibited by OPA-glycine-NAC isoindole derivatives. Additionally, the oxidative current peak at +0.8 V decreases over time, but another oxidation peak appears near +0.5 V and progressively increases, as presented in the insert diagram of Figure 7. The time-dependent minor oxidative current at around +0.5 V may be contributed by the electroactive OPA-glycine substance formed by the Schiff base. It was confirmed by our previous work [34] that the OPA-glycine compound had only 2% of normalized sensitivity compared with the OPA-glycine-NAC isoindole derivate. The oxidative current is significantly predominated by OPA-glycine-NAC isoindole derivate but insignificantly affected by OPA-glycine and almost negligible in a short period (<30 s).

An interval of 20 to 30 s was revealed by the results in Figure 7 to be the optimal test time for the OPA sensor, which was used in further experiments.

### 3.7. Comparison between the OPA Sensor and Amperometry with Bare SPCEs in a Batch Test

**Figure 8:**
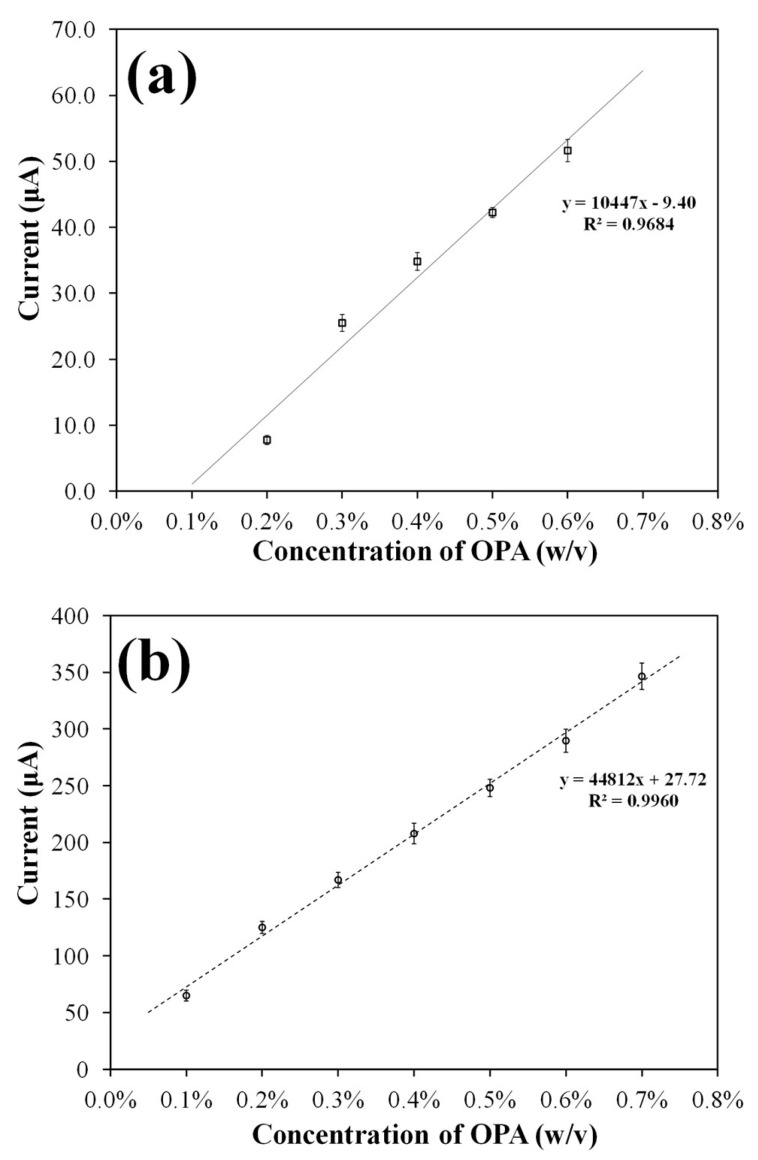
Calibration curve comparison between the OPA sensor and amperometry with SPCEs. (**a**) the PA hydrogel-modified SPCE strips were conditioned by exposing them to 5 µL of OPA for 20 s before applying +0.8 V vs. Ag/AgCl. The sensitivity was found to be about 3.3 μA/cm^2^ mM, and the variation coefficient was less than 10% (n = 3). (**b**) OPA detection was carried out using bare SPEC strips in batch (0.1 M PBS, pH 7.6) containing 50 mM glycine and 30 mM NAC. The electrodes were subjected to the same potential as in condition (**a**), and the sensitivity was about 14.2 μA/cm^2^ mM, with the variation coefficient being less than 3% (n = 3).

The performance of OPA sensors modified with a hydrogel containing glycine and NAC was determined, and unmodified SPCE strips were used in batches as a reference approach for clinically practical disinfectant concentrations (0.2–0.6% OPA). As shown in Figure 8a, a good correlation coefficient (0.9684), sensitivity (3.3 μA/cm^2^ mM), and a coefficient of variation of 10% (N = 3) in the linear detection range (0.2–0.6% OPA) were observed for the OPA sensor, which is consistent with the requirements of clinical disinfecting applications.

Normalized sensitivities of 3.3 μA/cm^2^ mM and 14.16 μA/cm^2^ mM were obtained for the OPA sensor measuring a drop of analyte and for the bare SPCE electrochemistry in a solution containing reagent, respectively. Furthermore, coefficients of variation of 10% and 3% were obtained for the OPA sensor and bare SPCE electroanalysis, respectively. Despite slight differences in performances (sensitivity and coefficient of variation) compared to the directive electroanalysis of OPA by SPCE in the batch (Figure 8b), the developed OPA sensor still complied with the design objectives of convenient test procedures and the indicated concentration range. Minor intrinsic issues for gel-modified electrodes, such as slightly poor sensitivity and coefficient of variation, were observed and could be improved and optimized in future mass-production phases.

### 3.8. Performance Verification by Comparison with Cidex-OPA and Reference OPA

A prototype of the potentiostat was developed (Figure S1 in the supporting information) to replace the electrochemical potentiostat. Its output signals were transferred to digital forms and utilized in the following works. The sensing performance of this electrochemical meter was verified by comparing it with a commercial potentiostat, and it was found to be hardware compatible with SPCE electrodes.

**Figure 9:**
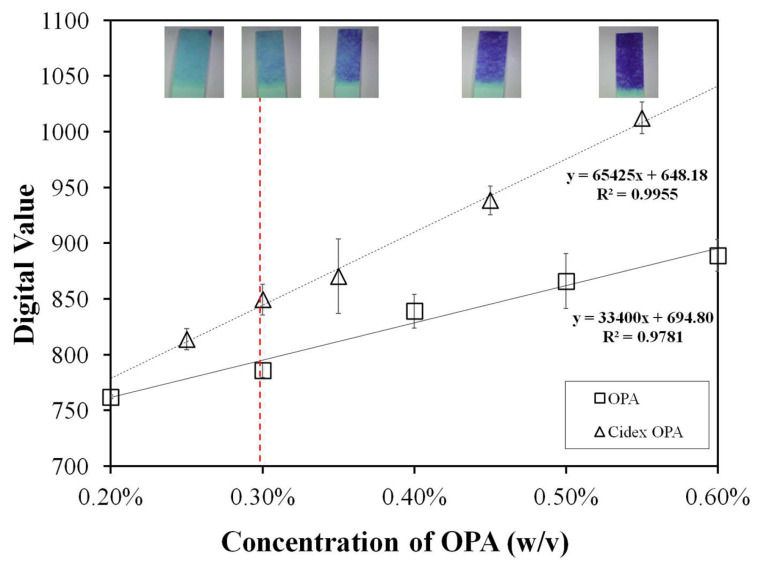
Calibration curve comparison of the OPA sensor between Cidex-OPA (△) and reference OPA (□). The hydrogel-modified SPCE strips were conditioned by exposing them to 5 µL of OPA for 20 s before being applied with +0.8 V vs. Ag/AgCl. The digital amperometric output was recorded using a homemade potentiostat prototype based on the LMP91000EVM module. The color of Cidex-OPA test strip indicators corresponding to Cidex-OPA test concentrations was shown in the upper photographs. The minimum effective concentration (MEC) of Cidex-OPA disinfectant was indicated by the red dot line.

The performance comparison of the developed OPA sensor to a commercially available disinfectant, Cidex–OPA, and a reference OPA solution for feasibility verification of the OPA sensor with a meter used in clinics was conducted. As shown in Figure 9, a slightly better correlation coefficient of the linear calibration curve to Cidex-OPA (0.9955) than to reference OPA (0.9781) at practical concentration ranges (0.2–0.6%) was obtained. Both variation coefficients of the OPA sensor determining the reference OPA and Cidex-OPA solutions were about 3%, indicating that the modification quality of the hydrogel on the electrodes had been improved by the sensor and that the analytical variation was at a similar level to that of the SPCE electrodes in batch tests (Figure 8b). Furthermore, almost twice the sensitivity to Cidex-OPA (18.28 μA/cm^2^ mM) of reference OPA (9.32 μA/cm^2^ mM) was observed by the sensor. The higher sensitivity of Cidex-OPA could be attributed to other electro-active substances contained within the commercially available disinfectant reagents, as confirmed by cyclic voltammetry supported by supplementary information (Figure S3). A distinct oxidation current was observed between 0.35 V and 0.5 V in the Cidex-OPA solution but no significant response was found between 0 V and 0.8 V in the reference OPA. This result indicates that the commercially available disinfectant Cidex-OPA formula contains other electroactive substance(s), such as benzotriazole and D&C Green Dye #5, which were mentioned in a use instruction claimed by the manufacturer. These electrochemical characteristics of benzotriazole are also supported by another report [35]. Reductive additives are generally adopted in OPA disinfectant solutions to prolong storage time and maintain OPA quality, which is a habitual art in commercial products because the OPA reagent oxidizes at room temperature, making its disinfecting quality hard to maintain. Although the developed OPA sensor is over-responsive, it does not affect sensor availability in experimental conditions by allowing calibration for a specific disinfectant OPA formula.

The colorations of test strips for different concentrations of Cidex-OPA disinfectant are shown on top of Figure 9, as determined by following the manufacturer’s instruction manual. Significant color differences are observed in two extreme conditions (initial 0.55% and final 0.25%), but effective color discrimination is difficult to obtain with the naked eye around MEC (0.3%). The poor precision of the semi-quantitative principle of chemical test strips increases infection risk and the management cost of verification of disinfectant concentrations.

The color change of test strips according to the specification of disinfectant concentration measured by commercially available chemical test strips should be determined at 90 s. However, accurately choosing the color interpretation of the test strip at the required moment in the clinical process is difficult. Test strip indications at different time moments for various OPA concentrations near MEC (0.25–0.35%) are listed in Table S1 of the supplementary material. The color of each test strip fades with time, and the color fades more in lower concentration conditions. Therefore, misjudgment by the user may be caused, especially in the disinfectant near the MEC of 0.3% for OPA. The Cidex-OPA test strip based on the semi-quantitative method depends on naked-eye visual judgments and is strongly time-dependent for color presentation. In contrast, the advanced OPA sensor can obtain the measured value at a fixed time using presets in electronic instruments. Additionally, the sensor has an excellent linear detection range from 0.25% to 0.55% of Cidex OPA and has sufficient sensitivity around the MEC of OPA disinfectants.

## 4. Discussion

GA, OPA, peracetic acid, and ethylene oxide have been approved by the US Food and Drug Administration (FDA) as reagents that can deactivate pathogens to avoid cross-infection and disease transmission, with high-level disinfection required to meet the 6-log10 tuberculosis mortality rate target in a short period. GA and OPA are currently the most commonly used endoscope disinfectants because they cause less structural damage to instruments than other disinfectants. However, severe irritation and/or injury to the respiratory tract, blood, and tissues can be caused by GA, affecting the health of patients and medical professionals engaged in the disinfection procedure [8,9,10]. OPA has gradually replaced GA as the mainstream disinfectant, based on considerations of patient health, occupational safety and health, and disinfection performance in hospitals. However, OPA has a slightly higher cost than GA, which increases the barrier to adoption by institute administrations. A previous report showed that the concentration of OPA fell with each successive disinfection in an automated endoscope reprocessor (AER), as studied by high-performance liquid chromatography (HPLC), and declined significantly after about 50 sterilization cycles [15]. This result indicates that the OPA concentration must be ensured (verified) before each disinfection process to confirm that the disinfectant concentration meets the MEC of OPA (0.3%).

According to the conservative risk management strategy of infection control, all clinical professional associations and product manufacturers recommend that OPA solution be tested before each usage with indicator (test) strips to maintain disinfecting effectiveness. Therefore, the MEC value should be maximized, and the OPA disinfectant solution should be replaced early by the clinical system to comply with those requirements and improve quality control (eliminate false negatives). However, obstacles to adopting safe OPA methods are created by financial pressure from expensive OPA disinfectant reagents [16] and OPA indicator strips.

**Figure 10:**
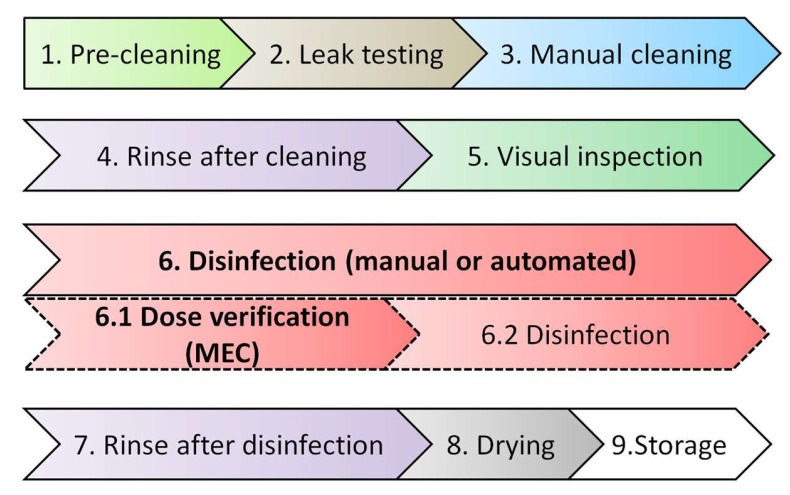
Scheme of endoscope reprocessing steps. Step 1: Pre-cleaning removes organic material and decreases the bioburden. Step 2: Leak testing detects damage to the endoscope’s interior channels and exterior surfaces. Step 3: The most critical step in removing the microbial burden from an endoscope. Step 4: Remove residual debris and detergent with de-ionized water. Step 5: Use magnification and adequate lighting to assist in visual inspection to ensure the endoscope is visibly clean. Step 6: Disinfectants must be tested to ensure they remain above their MEC. Step 7: Thoroughly rinse all surfaces of parts with de-ionized water. Step 8: Flushed with 70% to 90% ethyl or isopropyl alcohol before drying with pressurized, filtered air. Step 9: Endoscopes must be stored in a clean, well-ventilated, and dust-free area. Adopted and rewritten from ‘Standards of Infection Prevention in Reprocessing Flexible Gastrointestinal Endoscopes’, The Society of Gastroenterology Nurses and Associates.

The objective of the study was to develop a compact and simple quantitative OPA sensor that could substitute traditional OPA strips and be process-compatible with routine clinical disinfection operations. The convenience of the available OPA test strips based on dry chemistry on the market had attracted clinical practices. An ideal design requirement for the prototype is a one-step test procedure, similar to the dry chemistry-based OPA test strips. Although the wet chemistry-based OPA test (Figure 8b) performed better than the hydrogel-based OPA sensor (Figure 8a), its fatal flaw in cumbersome procedure and consumption of reagents, as well as samples, limits its potential for clinical practices. The sensitivity of the test strip was minor, but precision was critical for the specific detection range (0.2–0.6%) of OPA in this study. Hydrogels were found to be ideal for encapsulating drugs, proteins, and other biomolecules in biosensing, as they can maintain their shape while absorbing large amounts of water or aqueous media [36]. PA-hydrogel was introduced into the construction of an OPA sensor to meet the needs of clinical users for one-drop and one-step testing in this study. The electrochemical OPA sensors, based on PA as an alkaline, semi-solid, gel-like material containing reagents, were found to exhibit ease of use, similar to quasi-dry chemistry, making them suitable for clinical applications. The developed OPA sensor is designed to provide measurements equivalent to traditional OPA strips used in hospitals. It will be utilized in the dose-verification step (step 6.1) of the endoscope reprocessing procedure, as shown in Figure 10. The OPA sensor is inserted into a meter, similar to a glucose meter, and its sensing area is immersed in the OPA disinfectant solution in the AER/container, or a small sample of the disinfectant solution is applied to the sensing area. The meter’s digital display will then provide the OPA dose level, which can be used to confirm if the dose level exceeds the MEC required for effective disinfection.

Although important in biosensors, the selectivity assessment was not considered crucial and was not defined as a pending issue in this study. The OPA measurements are expected to be made in the hospital in a way called dose verification, which ensures that OPA solutions have no other chemicals or contaminants, as shown in step 6.1 of Figure 10. As part of regular high-level disinfection processes in clinics, medical devices such as endoscopes must be thoroughly cleaned (step 3) and rinsed (step 4) with de-ionized water and undergo strict inspection (step 5) before undergoing OPA disinfection (step 6). In the specific application scenario, both the object to be disinfected and the disinfectant solution are free from contaminants. Even though GA, another commonly used disinfectant, may pose potential cross-contamination risks when alternatively used in the same AER as OPA, the situation is infrequent and discouraged. Therefore, the concern of follow-up interference can be mitigated.

The design and development of an alternative product with better resolution around MEC of OPA (0.3%) for the dose-verification process and simple one-step operations such as dry chemistry-based test strips were the focus of this work. For the specific need of dose verification, precision was deemed more crucial than detection limit and detection range in assessing the sensor’s performance. Both the detection limit and detection range were minor for system development in this study because the OPA dose range specification was 0.2–0.6%, as defined by clinical need, which does not challenge the lower detection limit and the detection range. The customary emphasis on analysis and measurement is on pursuing excellent detection limits and sensitivity, but this study was a mission-oriented work focusing on precision and process compatibility. The precision of the OPA sensor could be significantly improved by pre-treating SPCEs (Figure 6 and Table 1). Furthermore, the precision of the sensor will not be significantly degraded by the coated hydrogel. Based on the practical considerations of dose verification in clinics, this study omits the assessment of the sensor’s performance when the OPA concentration is lower than 0.2% (including the background value of 0.0%) and higher than 0.6%. If the application scenario is low-concentration OPA measurements in the future, this approach can be adopted again to extend the development of sensors and their applicable range. Moreover, for the specific need for OPA dose verification in the clinic disinfecting process, the specificity of the sensor could be omitted because medical devices such as endoscopes must be thoroughly cleaned and inspected before the disinfecting procedure, so contamination is almost negligible.

In summary, our developed quantitative OPA sensor with good detection precision and accuracy is available and meets the clinical verification needs of OPA MEC.

## 5. Conclusions

An innovative and cost-effective approach for the quantification of OPA through electrochemical sensors based on SPCEs modified with hydrogel is presented in this study. The SPCEs were optimally modified by the PA hydrogel film, which contains 50 mM glycine and 30 mM NAC in 0.1 M PBS (pH 7.0). The sensor’s response was characterized to obtain the shortest possible measurement time (20–30 s). Analytical results show that the developed OPA sensor meets the design requirements for convenient test procedures and usability as an indicator in the standard dose range for application (0.2% to 0.6%). The OPA sensor was validated in commercial Cidex-OPA, and good linearity (r^2^ = 0.9955) was observed over a practical concentration range used in clinical applications. Additionally, the advanced OPA sensor has sufficient sensitivity (18.28 μA/cm^2^ mM) around the MEC (0.3%) of Cidex-OPA disinfectant. Consequently, the developed OPA sensor is superior to current semi-quantitative indicator strips, providing greater precision, reliability, and lower cost. This makes it a promising tool for the simple and reliable quantitative verification of OPA disinfectant concentrations used in clinical applications.

## Figures and Tables

**Table 1:** Repeatability and reproducibility of the electrochemical response of SPCEs after various pretreated processes. Amperometric responses of SPCEs in 4 mM K_3_[Fe(CN)_6_]/1 M KCl at +0.5 V vs. Ag/AgCl.

Pre-Treatmzent Methods	Repeatability ^a^		Reproducibility ^b^	
	Mean Response (μA) (Mean)	CV (%)	Mean Response (μA) (Mean ± S.D.)	CV (%)
Activated in 0.05 M PBS at +1.2 V for 2 min	36.4	4–31%	39.6 ± 8.70	21%
Activated in Sat. Na_2_CO_3_ at +1.2 V for 5 min	49.8	1–3.7%	51.2 ± 4.17	8%
Soaked in 3 M NaOH for 1 h, and then activated in 0.5 M NaOH at +1.2 V for 20 s	43.9	6–15%	41.1 ± 6.85	16%

^a^: The data represent the average values of three measurements from the same electrode (n = 3). ^b^: The data represent the average values of 15 measurements from five-independent electrodes (N = 5).

## Data Availability

Not applicable.

## Supplementary Materials

The following supporting information can be downloaded at: https://www.mdpi.com/article/10.3390/bios13040485/s1, Figure S1: Schematic of the miniaturized integrated potentiostat for determination of *o*-phthalaldehyde; Figure S2: Calibration curves of various resistances of the transimpedance amplifier in potentiostat; Figure S3: Cyclic voltammograms of 0.1% reference OPA and 0.1% Cidex-OPA in 0.1 M PBS (pH 7.0) with bare SPCEs; Table S1: Test strip indications at different time moments for various OPA concentrations near MEC (0.25–0.35%).
