# Supplementary material for: An Electrochemical o-Phthalaldehyde Sensor Using a Modified Disposable Screen-Printed Electrode with Polyacrylate Hydrogel for Concentration Verification of Clinical Disinfectant
Source: Biosensors (Basel). 2023 Apr 17;13(4):485. doi: 10.3390/bios13040485 (PMC10136433; doi:10.3390/bios13040485)
Supplement: Supplementary file 1 [file biosensors-13-00485-s001.zip › biosensors-2307779-supplementary.pdf]

biosensors

MDPI

Supporting Information

An Electrochemical *o*-phthalaldehyde Sensor Using a Modified Disposable Screen-Printed Electrode with Polyacrylate Hydrogel for Concentration Verification of Clinical Disinfectant

Richie L. C. Chen [1], Bo-Chuan Hsieh [1], Jia-Sin Lin [1] and Tzong-Jih Cheng [1,2,*]

1 Department of Biomechatronics Engineering, College of Bio-Resources and Agriculture, National Taiwan University, Taipei 10617, Taiwan; rlcchen@ntu.edu.tw (R.L.C.C.); hsiehpc@ntu.edu.tw (B.-C.H.); r03631020@ntu.edu.tw (J.-S.L.)
2 Department of Biomedical Engineering, National Taiwan University Hospital, College of Medicine, National Taiwan University, Taipei 10617, Taiwan
* Correspondence: tzongjih@ntu.edu.tw

Construction of a potentiostat prototype for hydrogel-modified SPCEs

An electrochemical sensor module (LMP91000EVM) purchased from Texas Instruments, which performs low-power electrochemical sensing and provides complete signal transmission from the sensor to a microcontroller. A microcontroller receives the module's digital voltage signal proportional to the sensing current. It can also be equipped with a Bluetooth module to develop a wireless chemical sensing system. In addition, the module can be programmed using the I^2C protocol interface for programmable amplifier selection. The main functions include 2-lead ground referred galvanic cell or 3-lead amperometric cell and a temperature sensor. Additionally, the appropriate signal amplification is programmed by the I^2C interface to set the optimal detection range and sensitivity. This sensor module is equipped with build-in seven transimpedance gains for internal programming adjustments, including 2.75 kΩ, 3.5 kΩ, 7 kΩ, 14 kΩ, 35 kΩ, 120 kΩ, and 350 kΩ.

In this study, the LMP91000EVM was programmed by an Arduino microcontroller, and the 3-lead amperometric cell was selected as the potentiostat. To verify the potentiostat function of LMP91000EVM module, ferrocyanide in 0.1 M phosphate buffer solution (pH 7.5) was used as reference solution to program values of the "Internal zero selection (Percentage of the source reference)" and of the "Selection of the Bias polarity" by the microcontroller for providing a sufficient oxidative potential (+0.6 V) of ferrocyanide between working electrode and reference electrode. Furthermore, the feedback resistance of transimpedance amplifier in the module was programmed to adjust the feedback resistance value of the amplifying circuit for fitting electrochemical response of the detection range of analyte concentration. These results of sensing performance were also confirmed by a commercial potentiostat (CH Instruments).

The sensing system is a hydrogel-modified electrochemical strip integrated with a handheld potentiostat to measure concentration of disinfectant OPA and to meet requirement of detection range of OPA in clinical applications. As shown in Fig. S1, the potentionstat module LMP91000EVM is controlled by the microcontroller Arduino and combined with a seven-segment display to display the concentration value and the LED light indicates the current status. In order to establish an OPA calibration curve for this system, OPA standard solutions (0.2%–0.6%) were analyzed with the home-made potentiostat. The OPA sensors were controlled to apply +0.8 V after 20 seconds of preconditioning, and digital outputs of amperometric response were recorded at the 5th seconds.

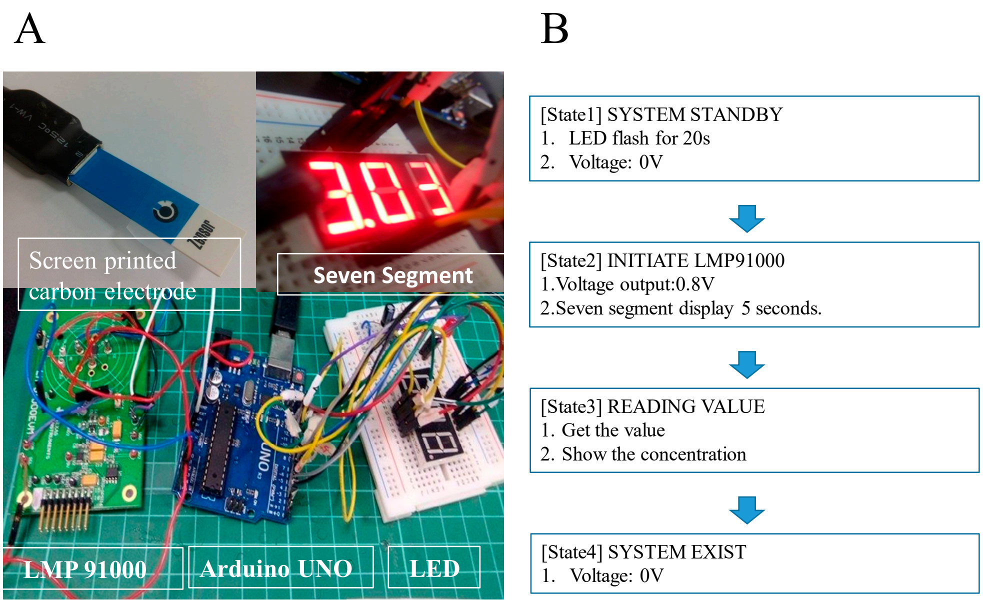

Figure S1. (**A**) Schematic of the miniaturized integrated potentiostat for determination of *o*-phthalaldehyde. (**B**) The main procedure of potentiostat LMP91000EVM.

Feasibility of the home-made potentiostat used in OPA sensors

The feedback resistance (2.75 kΩ ~ 350 kΩ) of transimpedance amplifier predominate magnification factor, and the bit-numbers (10-bits) of digital signal output preset system's precision as well as output range. Moreover, Detection range and sensitivity are proportional but inversely proportional to the feedback resistance, respectively. Therefore, proper feedback resistance used in the transimpedance amplifier of potentiostat is the key issue for trade-off between detection range and sensitivity of sensing system.

In the performance test of the hydrogel-modified electrochemical strip to OPA, the current response maximum value is about 60 μA in the detection range of 0.2%-0.6% (refer to Fig. 3(a)). This result was set as criteria of detection range for determining the feedback resistance of transimpedance amplifier in potentiostat. Figure S2 shows calibration curves for various feedback resistance of the transimpedance amplifier. The resistance value of 35 kΩ, satisfies the detection range required for OPA detection by the hydrogel-modified SPCE strip, and has a good sensitivity in the linear range of 0-100 μA. This work confirmed that the LMP91000EVM-based potentiostat prototype meets the detection range requirement of OPA determined by the developed OPA sensor.

In order to develop the OPA sensor system (meter), the Internal zero selection (Percentage of the source reference) and the Selection of the Bias polarity function of the potentiostat are programmed firstly, so that the potential difference between the working electrode and the reference electrode is +0.8 V to satisfy requirement of oxidative potential for the hydrogel-modified SPCE strips. Then the potentiostat and the hydrogel-modified SPCE strip were combined as an OPA sensing system to determine 0.2%.-0.6% OPA solutions. According to preliminary result above-mentioned, the 35 kΩ of feedback resistance was applied to the potentiostat, for measuring OPA, but its output signal exceeds the digital output upper-limit (2^{10}=1024) in the detention range of OPA. Furthermore, the resistance value was fine down-tuned to 14 kΩ, detection range of the system was sufficiently complied with specification of the OPA sensor and posed good

correlation coefficient (0.9781) in the linear range with a coefficient of variation of 3%. These results implied that the developed OPA sensing system integrated with a hydrogel-modified SPCE strip and prototype electrochemical meter can meet the demand for determination of OPA.

A

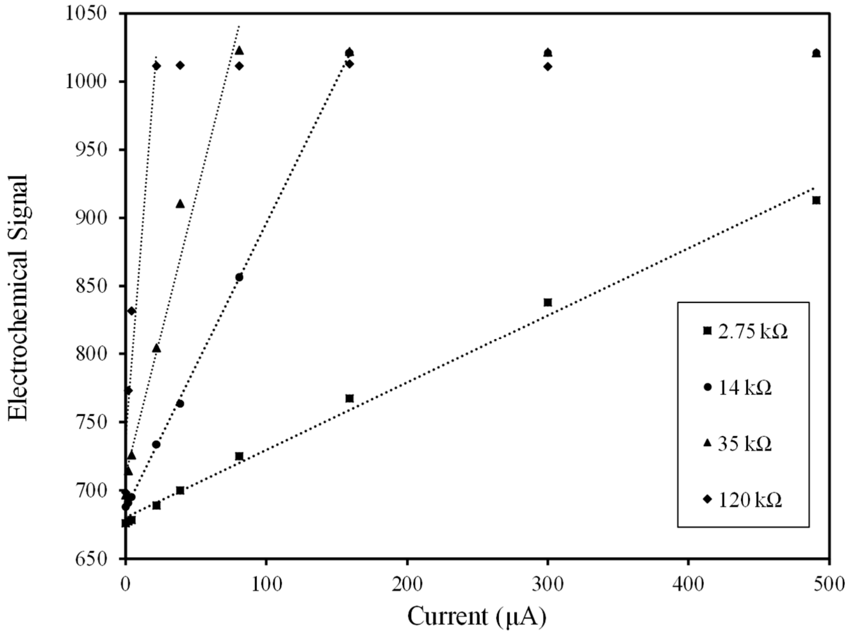

B

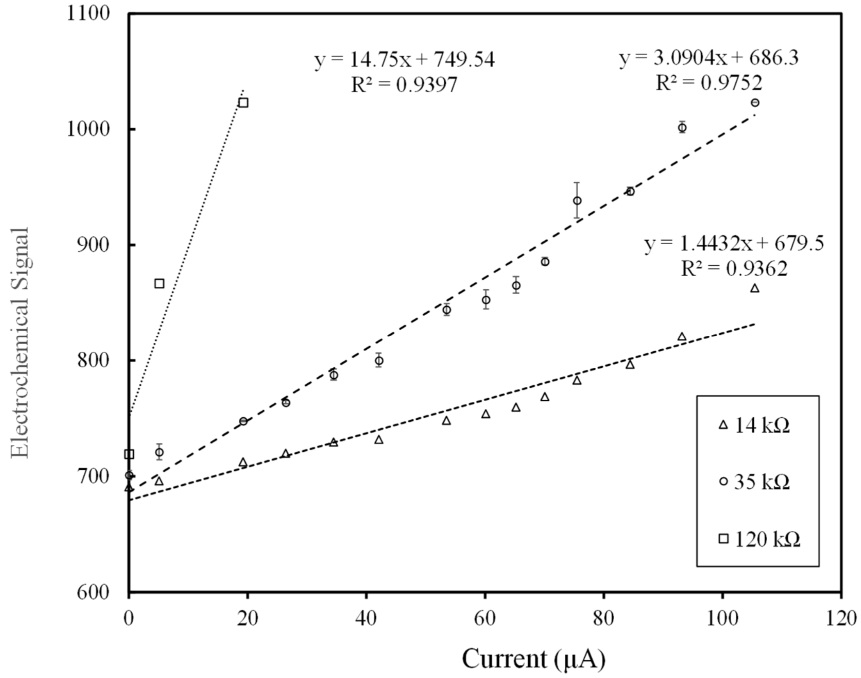

Figure S2. Calibration curves of various resistances of the transimpedances amplifier in potentiostat. Curve represents the amperometric response for determination of potassium *ferrocyanide* at +0.6 V by a potentiostat LMP91000EVM with bare SPCE strip. Current values range from (**A**) 0 μA to 500 μA. (**B**) 0 μA to 100 μA. Electrochemical signal: the data from digital values of the LMP91000EVM potemtiostat.

Electrochemical characteristics of Cidex-OPA and reference OPA

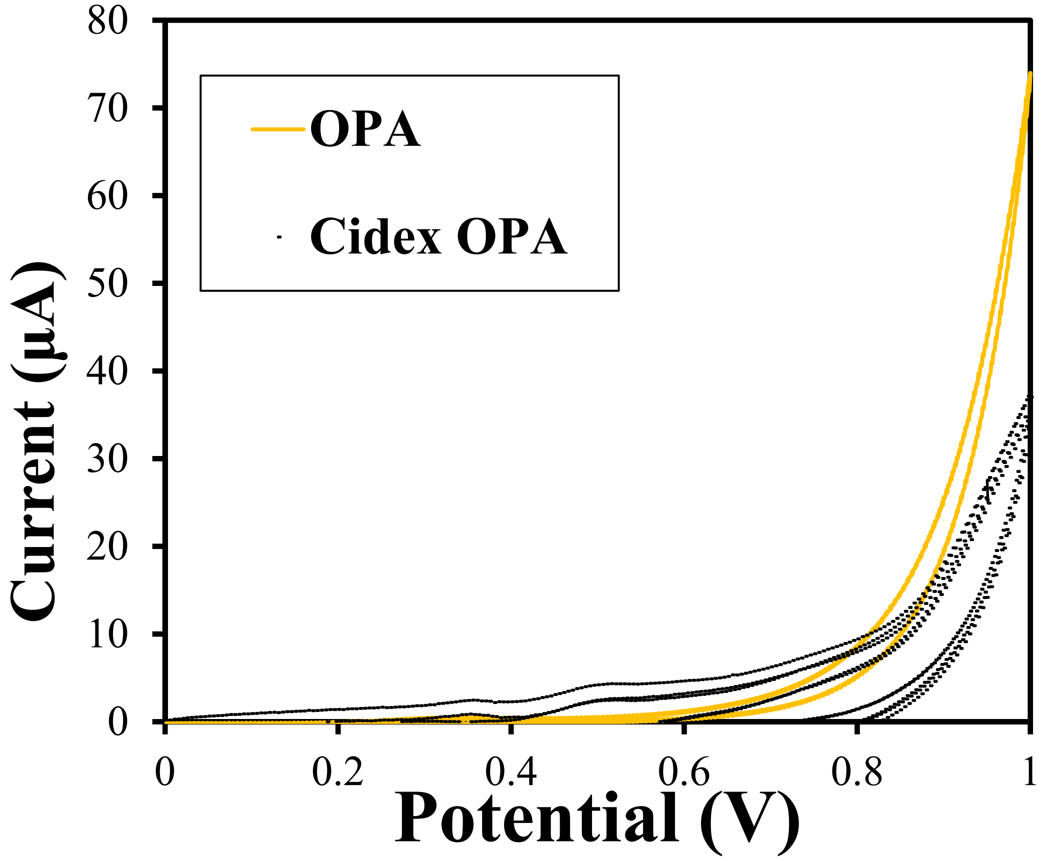

Figure S3. Cyclic voltammegrams of 0.1% OPA (solid line) and 0.1% Cidex-OPA (dash line) in 0.1 M PBS (pH 7.0) with bare SPCEs. Scan rate: 100 mV/s.

Table S1. Test strip indications at different time moments for various OPA concentrations nearby MEC (0.25%–0.35%).

Time (s) / OPA (w/v)	60	70	80	90	100	110	120
0.35%	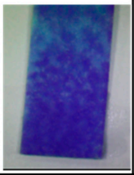	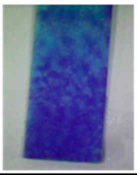	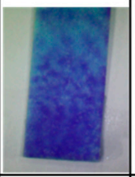	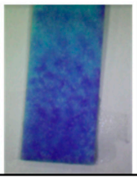	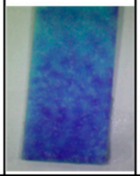	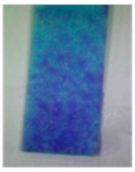	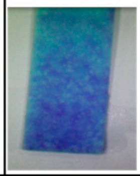
0.33%	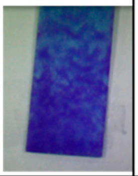	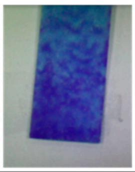	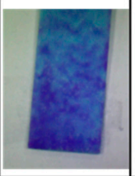	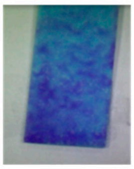	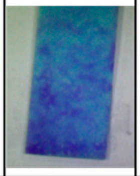	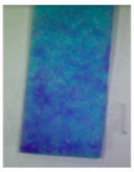	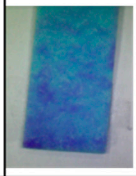
0.30%	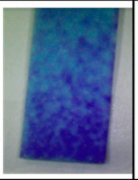	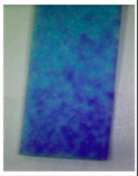	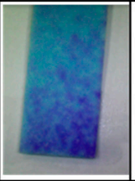	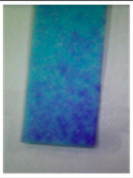	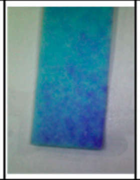	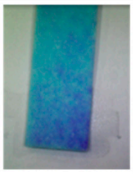	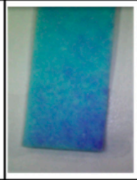
0.27%	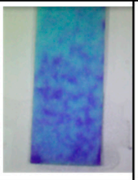	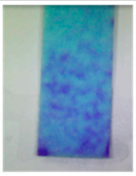	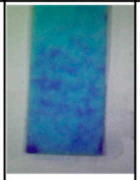	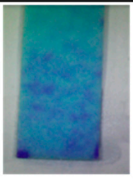	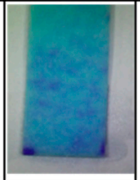	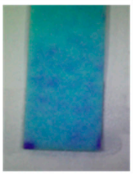	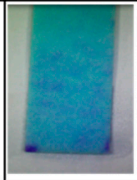
0.25%	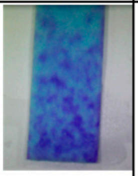	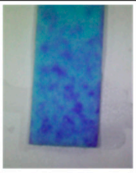	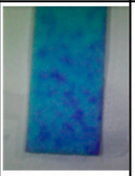	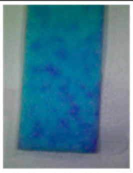	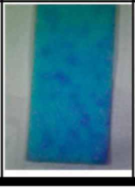	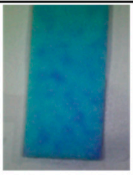	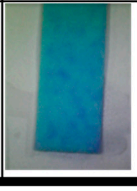
